# Enzyme stabilization and thermotolerance function of the intrinsically disordered LEA2 proteins from date palm

**DOI:** 10.1038/s41598-023-38426-w

**Published:** 2023-07-23

**Authors:** Mughair Abdul Aziz, Miloofer Sabeem, M. Sangeeta Kutty, Shafeeq Rahman, Maitha Khalfan Alneyadi, Alia Binghushoom Alkaabi, Eiman Saeed Almeqbali, Faical Brini, Ranjit Vijayan, Khaled Masmoudi

**Affiliations:** 1 Department of Integrative Agriculture, College of Agriculture and Veterinary Medicine, United Arab, Emirates University, Al‑Ain, Abu‑Dhabi, UAE; 2 Department of Vegetable Science, College of Agriculture, Kerala Agricultural University, Vellanikkara, Thrissur, 680656 India; 3 Biotechnology and Plant Improvement Laboratory, Centre of Biotechnology of Sfax (CBS)/ University of Sfax, Sfax, Tunisia; 4 Department of Biology, College of Science, United Arab Emirates University, Al‑Ain, Abu‑Dhabi, UAE

**Keywords:** Biotechnology, Computational biology and bioinformatics, Molecular biology, Plant sciences

## Abstract

In date palm, the *LEA2* genes are of abundance with sixty-two members that are nearly all ubiquitous. However, their functions and interactions with potential target molecules are largely unexplored. In this study, five date palm LEA2 genes, *PdLEA2.2*, *PdLEA2.3*, *PdLEA2.4*, *PdLEA2.6*, and *PdLEA2.7* were cloned, sequenced, and three of them, *PdLEA2.2*, *PdLEA2.3*, and *PdLEA2.4* were functionally characterized for their effects on the thermostability of two distinct enzymes, lactate dehydrogenase (LDH) and β-glucosidase (bglG) in vitro. Overall, PdLEA2.3 and PdLEA2.4 were moderately hydrophilic, PdLEA2.7 was slightly hydrophobic, and PdLEA2.2 and PdLEA2.6 were neither. Sequence and structure prediction indicated the presence of a stretch of hydrophobic residues near the N-terminus that could potentially form a transmembrane helix in PdLEA2.2, PdLEA2.4, PdLEA2.6 and PdLEA2.7. In addition to the transmembrane helix, secondary and tertiary structures prediction showed the presence of a disordered region followed by a stacked β-sheet region in all the PdLEA2 proteins. Moreover, three purified recombinant PdLEA2 proteins were produced in vitro*,* and their presence in the LDH enzymatic reaction enhanced the activity and reduced the aggregate formation of LDH under the heat stress. In the bglG enzymatic assays, PdLEA2 proteins further displayed their capacity to preserve and stabilize the bglG enzymatic activity.

## Introduction

Plants have evolved complex regulatory pathways to counter the effects of adverse climatic conditions. The mechanisms to improve abiotic stress tolerance are largely dependent on protein molecules that directly function and regulate various plant physiological processes and signaling pathways. The late embryogenesis abundant (LEA) protein gene family is a group of functional proteins that protect and reduce plant cell damages under abiotic stress conditions^1^. These proteins are disordered in their structure and characterized by repeated motifs^2^. Based on the conserved amino acid sequence motifs, LEA proteins were classified into eight distinct groups, with LEA2 proteins being the most predominant group in plants. Primarily, LEA2 proteins were found in large quantities in mature seeds of *Gossypium hirsutum*^3^, and were ubiquitously expressed in flowering and non-flowering plants^4^. They mainly accumulate in the late phases of seed development and in vegetative tissues for plant responses to environmental constraints^5^. LEA2 proteins are immensely hydrophilic and characterized as intrinsically disordered proteins (IDPs) that can vary their conformation in response to the changes in the ambient microenvironment^6^. Among the LEA2 proteins sub-family, dehydrins (DHNs) are a well-known biochemical group that is mainly composed of high proportion of charged and polar amino acids, and low fraction of hydrophobic and non-polar residues^7^.

LEA2 proteins are broadly involved in plants physiological responses to improve abiotic stress tolerance. Overexpression of *Triticum aestivum* L., *TaLEA2-1* in wheat enhanced their root growth, plant height, and led to higher catalase activity in comparison to the wild type seedlings. *TaLEA2-1* conferred improved salinity tolerance in *TaLEA2-1* transgenic wheat plants^5^. Furthermore, in a recent study, *LEA2* gene, *PtrDHN-3*, from *Populus trichocarpa* was found to play an essential role in salt and drought stress tolerance8. It was observed that the overexpression of *PtrDHN-3* increased the salt tolerance of transgenic yeast, and improved the germination rate, fresh weight, and chlorophyll content of transgenic *Arabidopsis thaliana* plants under salt stress^8^. In addition, the Arabidopsis plants transformed with cotton *LEA2* genes displayed higher growth under drought stress compared to the wild type^9^. Moreover, in maize a KS-type DHN gene, *ZmDHN13*, was isolated and its overexpression in transgenic tobacco plants enhanced the oxidative stress tolerance^10^. In another study, overexpression of *Prunus mume LEA2* gene in transformed tobacco and *Escherichia coli* improved cold stress tolerance^11^. Furthermore, overexpression of two *A. thaliana* DREs, *AtDREB1A* or *AtDREB2A*, resulted in an induction of cold stress interlinked genes of LEA2 proteins, such as the *rd29A* and *COR47*^12^. In the putative promoters of *LEA2* genes of upland cotton, *G. hirsutum*, several abiotic stress-related cis-elements were found. It included MYBCORE, ABRELATERD1, ABRE-like sequence and ACGTATERD1 elements which are known to have a functional role in abiotic stresses^13,14^. The presence of these stress promoter elements strongly supports the role of LEA2 proteins in enhancing abiotic stress tolerance in plants growing under hostile climates.

In the resurrection plant, *Craterostigma plantagineum*, several LEA mRNA transcripts and proteins have been identified during a cycle of desiccation stress^15^. Similarly, to gain insights into the *Phoenix dactylifera*, a woody extremophile plant that thrives under harsh environmental conditions, an RNA-Seq analysis was performed to understand the date palm ABA signaling pathway in response to drought stress^16^. Date palm’s pinnae was treated with the hormone ABA that resulted in the expression of 153 differentially expressed genes (DEGs)^16^. Among the highlighted genes, *LEA* genes were reported to be upregulated along with phosphatases from the PP2C family, ATP binding cassette (ABC) transporters, and the guard cell transcription factor MYB74. In addition, a whole genome sequencing of date palm was performed and *LEA2* genes were found abundantly present in date palm’s genome assembly^17^. It was found that *LEA2* genes consisted of sixty-two variants in date palm^17^. Moreover, high expression of *LEA2* genes were reported in date palm inoculated with *Piriformospora indica* under salinity stress^18^. These findings argue in favor of *LEA2* genes involvement in the date palm’s abiotic stress tolerance mechanisms.

Although several molecular and physiological analyses of stress related genes in *P. dactylifera* have been performed, the functional characterization of *P. dactylifera LEA2* (*PdLEA2*) genes is still obscure^19^. However, it is believed that LEA2 proteins are highly flexible unstructured proteins that are able to function as chaperones and interact with several partner molecules such as proteins, membranes, nucleic acids, and metal ions^20^. A recent study annotated the functional properties of LEA2 proteins, which included their roles in protecting membranes, stabilizing macromolecules, supporting in free radical scavenging, and acting as antioxidants for alleviating the oxidative damages caused to plants under the abiotic stress conditions^6^. Similar to LEA2 proteins, major heat-shock proteins (hsp) have some kind of related roles in solving the problem of misfolding and aggregation, as well as their role as chaperones. Thus, LEA2 proteins play crucial roles in protecting other protein molecules and enzymes from aggregation and stabilize their activities under several hostile treatment conditions. Nevertheless, few functional enzymatic assays have been developed for examining the protective role of LEA2 proteins on enzyme activities. Moreover, it is observed that majority of the industrial enzymes are degraded due to the extreme temperature in the processing conditions, during which the enzyme needs to be preserved.

Based on the functional properties of LEA2 proteins, they can be utilized for the stabilization and preservation of enzymatic activities from heat during industrial processes for a longer period. In relation to this, it is necessary to investigate the role of LEA2 proteins in preserving the thermosensitivity of lactate dehydrogenase (LDH) and β-glucosidase (bglG) enzymes. LDH enzyme acts as a redox cofactor by utilizing the NADH/NAD^+^ pair for catalyzing the interconversion of pyruvate (oxo acid) and lactate (alpha-hydroxy acid)^21^. It is used for the conversion of plant pyruvate into lactic acid under anaerobic conditions. While bglG is a cellulolytic enzyme that is employed in the degradation of cellulosic biomass. It is involved in the hydrolysis of carbohydrates such as starch, glycogen, and their disaccharides derivatives into their monomers^22^. Both of these enzymes are used on a large scale for industrial and biotechnological applications.

Thus, the objective of the current study was to examine and characterize the role of *P. dactylifera* LEA2 proteins on enzymes thermotolerance. The present study emphasized on the similarities and dissimilarities between the five PdLEA2 proteins in terms of their physicochemical characteristics, amino acid constituents, disorder propensity, preserved structural motifs, and predicted their secondary and tertiary structure. Furthermore, PdLEA2 recombinant proteins were produced and their protective role on LDH and bglG enzymatic activities and stabilities under heat stress condition was elucidated. The findings of the present study will be the first breakthrough to identify the chaperone property of date palm LEA2 proteins in protein-enzyme interaction. It will build a pathway for identification of LEA2 proteins integral partners and target components in the cell, providing expanded perception into their protective phenomenon for crop stress tolerance.

## Results

### 1Isolation and physicochemical analysis of PdLEA2 sequences

The PdLEA2 genes were mapped to different chromosomes in date palm. Based on a BLAST search against the Barhee BC4 date palm genome assembly (GCF_009389715.1), *PdLEA2.2* was not assigned to any chromosome, while the *PdLEA2.3, PdLEA2.4, PdLEA2.6*, and *PdLEA2.7* were distributed in the chromosomes 7, 14, 3, and 2, respectively. The mRNA sequences were deposited in NCBI GenBank and the mRNA and protein accessions are provided in Table S1.

**Figure 1:**
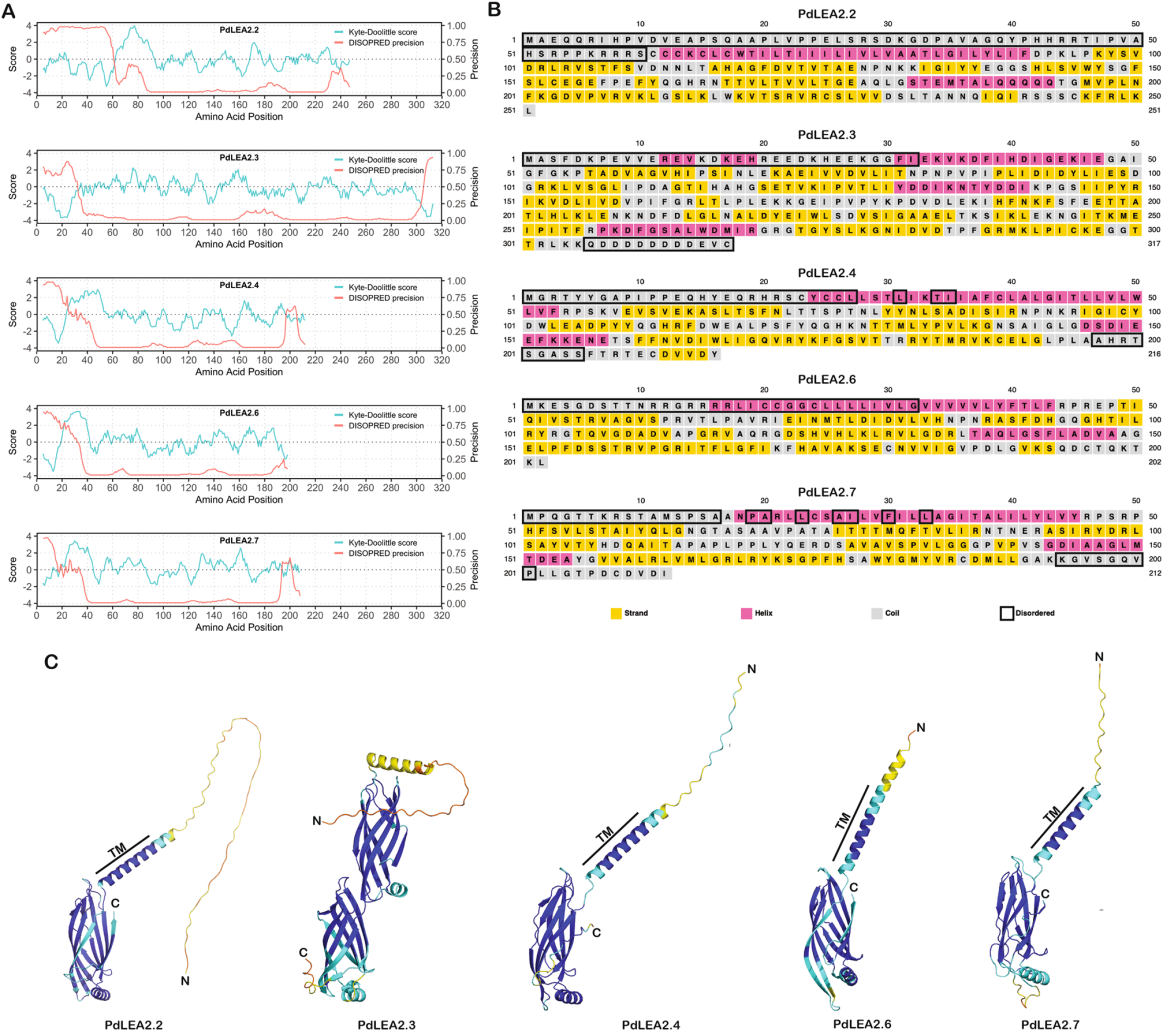
Structural characteristics of PdLEA2 proteins and their hydropathy. (**A**) Kyte-Doolittle hydropathy plots of PdLEA proteins (ProtScale, ExPASy). The PdLEA2.2, PdLEA2.3, and PdLEA2.4 proteins display almost identical hydropathy plots with certain variations at the N- and C- terminal, in contrast to PdLEA2.6 and PdLEA2.7 that possesses similar hydropathy plots. Disorder propensity of PdLEA2 proteins assessed using DISPORED is shown in green color. The default cutoff of 0.5 was used to define regions that are disordered. (**B**) Secondary structure prediction of PdLEA2 proteins using PSIPRED. The predicted α-helix region is colored pink, β-strand as yellow and random coil as gray. The disordered regions predicted by the DISOPRED is marked with black rectangles. (**C**) Three-dimensional structural model of PdLEA2 developed using AlphaFold2. The proteins are shown in cartoon representation and colored based on the pLDDT score, where blue: pLDDT > 90; cyan: 70 < pLDDT < 90; yellow: 50 < pLDDT < 90; orange: pLDDT < 50. The N- and C-termini of the proteins and the transmembrane (TM) region predicted by MEMSTAT are marked.

The PdLEA2 proteins predicted molecular weights ranged from 22.04 to 35.43 kDa, with PdLEA2.3 having the highest molecular weight of 35.43 kDa, with 317 amino acids, and PdLEA2.6 having the lowest molecular weight of 22.04 kDa, with 202 amino acids (Table 1). The physicochemical properties analysis revealed that most of the isolated PdLEA2 proteins had relatively high isoelectric points (pI > 7) (Table 1), indicating that the PdLEA2 proteins were mostly basic. The calculated GRAVY index (Table 1), an indication of the average hydrophobicity of the protein, showed that PdLEA2.7 was slightly hydrophobic (> 0), PdLEA2.3 and PdLEA2.4 were moderately hydrophilic (< 0), and PdLEA2.2 and PdLEA2.6 had a score close to 0. However, expanding this to the residue level, the Kyte-Doolittle plots (Fig. 1A) indicated the existence of stretches of highly hydrophobicity regions near the N-terminus of PdLEA2.2, PdLEA2.4, PdLEA2.6 and PdLEA2.7. The PdLEA2.2, PdLEA2.4, PdLEA2.6, and PdLEA2.7 were classified as unstable proteins with instability index of 49.27, 43.09, 41.89, and 41.83, respectively. Whereas PdLEA2.3 was quite stable with an instability index of 21.6. The aliphatic index of the five PdLEA2 proteins was high and ranged between 84.4 to 105.5, which indicated that PdLEA2 proteins are thermostable over a broad range of temperatures.

**Table 1:** Physicochemical properties of PdLEA2 proteins.

PdLEA2 proteins	Number of amino acids	MW	Theoretical pI	Instability index	Aliphatic index	GRAVY
PdLEA2.2	251	27.88 kDa	9.33	49.27	94.30	-0.092
PdLEA2.3	317	35.43 kDa	4.85	21.60	96.81	-0.368
PdLEA2.4	216	24.73 kDa	8.11	43.09	84.40	-0.19
PdLEA2.6	202	22.04 kDa	9.96	41.89	105.54	0.085
PdLEA2.7	212	22.55 kDa	9.22	41.83	101.27	0.296

### Structural characteristics of PdLEA2 proteins

####  Secondary structure and disorder propensity

A high similarity was identified in the secondary structure and disorder propensity of the five PdLEA2 proteins. The prediction of PdLEA2 sequence composition secondary structure was performed using PSIPRED (Fig. 1B; Table 2). Evidently, the most predominant folded secondary structure is the β-strand, 38–49%, predicted with the highest level of confidence. Random coils were present and distributed throughout the entire sequence. However, a long stretch of coiled region was predicted at the N-terminal region of all PdLEA2 proteins, which increased the total composition of this state, and ranged between 31–45% of the structures.

**Table 2:** Secondary structural composition and transmembrane α-helix region of PdLEA2 proteins.

PdLEA2 proteins	α-helices (%)	β-helices (%)	Random coils (%)	MEMSTAT	DeepTMHMM	TOPCONS
PdLEA2.2	17	38	45	61–90	70–90	66–86
PdLEA2.3	15	44	41	150–165	74–83	-
PdLEA2.4	19	39	42	30–53	35–53	27–47
PdLEA2.6	20	49	31	17–41	22–40	19–39
PdLEA2.7	19	41	41	22–44	22–44	21–41

As LEA2 proteins are known to harbor intrinsically disordered regions, disorder propensity of PdLEA2 was predicted using DISOPRED. In all the PdLEA2 sequences, a disordered region of varying length was identified at the N-terminal region of the protein (Fig. 1A). Additionally, a disordered region was also identified near the C-terminal region of PdLEA2.3, PdLEA2.4, and PdLEA2.7. The disordered regions corresponding to PdLEA2 proteins are enclosed in black rectangles in Fig. 1B.

####  Three-dimensional structural model of PdLEA2 proteins

3D structures of PdLEA2 proteins were modelled using AlphaFold2, an artificial intelligence-based protein structure prediction system. The predicted structures of the five proteins are shown in Fig. 1C. As evident from the figures, in all proteins, the N-terminal region had a distinct disordered region for which a 3D structure could not be predicted. This is indicated by the low value of predicted local distance difference test (pLDDT) value, an indicator of the residue-level confidence in the predicted structure, in this region. Overall, structurally all five PdLEA2 proteins appear to have a conserved architecture consisting of a disordered N-terminal region, followed by an α-helix and a tertiary structure consisting of two stacked β-sheets. Notably, the stacked β-sheet structure is duplicated in the case of PdLEA2.3.

#### Transmembrane region

An overlapping region with high hydrophobicity (Fig. 1B) and helicity (Fig. 1A) was predicted in each of the PdLEA2 proteins following the predicted disordered region (Fig. 1B). Therefore, to assess if the PdLEA2 proteins could localize to the membrane, multiple transmembrane domain prediction tools—MEMSTAT, DeepTMHMM and TOPCONS—were used to predict transmembrane regions in PdLEA2 (Table 2). Interestingly, all of these predictors indicated that the hydrophobic helix identified above is potentially a transmembrane α-helix. The only discrepancy appears to be in PdLEA2.3, where there was no consensus between the results of the three predictors. Furthermore, the predictors suggested that the disordered N-terminal region of PdLEA2 proteins is intracellular, while the β-strand rich region resides outside the cell.

####  Conserved domains and protein motifs

Conserved domains in the PdLEA2 sequences were identified using CD-Search against NCBI’s Conserved Domain Database (CDD). PdLEA2.2, PdLEA2.4, PdLEA2.6 and PdLEA2.7 proteins were found to harbor one complete conserved domain of the LEA_2 superfamily. While, PdLEA2.3 was observed to have two Water Stress and Hypersensitive response (WHy) domain, which is also a member of the LEA_2 superfamily (Table 3). All identified conserved domains (pfam03168 and smart00769) are members of the cl12118 LEA_2 superfamily. Additionally, MEME identified the presence of 4 statistically significant conserved motifs within PdLEA2 sequences (Table 4). However, only one conserved motif with a consensus sequence of DVLIRNPN was shared by all the five sequences.

**Table 3:** Conserved domains of PdLEA2 proteins.

PdLEA2 proteins	Specific hit	Superfamily	Accession	Residues
PdLEA2.2	LEA_2	LEA_2 superfamily	pfam03168	123–225
PdLEA2.3	WHy	LEA_2 superfamily	smart00769	58–157
	WHy	LEA_2 superfamily	smart00769	184–283
PdLEA2.4	LEA_2	LEA_2 superfamily	cl12118	14–93
	LEA_2	LEA_2 superfamily	pfam03168	86–189
PdLEA2.6	LEA_2	LEA_2 superfamily	cl12118	20–202
PdLEA2.7	LEA_2	LEA_2 superfamily	pfam03168	83–186

**Table 4:** Specific amino acid motifs of PdLEA2 proteins.

Motif consensus sequence and logo	PdLEA2 proteins	Sequence	Start position
WYQGFRFCWEEFPEFYQGHKNTTM	PdLEA2.2	YYQGHRFDWEALPSFYQGHKNTTM	108
	PdLEA2.4	WYSGFSLCEGEFPEFYQGHRNTTV	146
DVLIRNPN	PdLEA2.2	DVLITNPN	77
	PdLEA2.3	DVLVHNPN	80
	PdLEA2.4	DISIRNPN	86
	PdLEA2.6	TVLIRNTN	83
	PdLEA2.7	TVTAENPN	123
RRRSCCC	PdLEA2.2	RRRSCCC	57
	PdLEA2.4	RHRSCYC	19
	PdLEA2.6	RRRLICC	15
RYKFGSLKSRRYTMRVRC	PdLEA2.2	RYKFGSVTTRRYTMRVKC	172
	PdLEA2.4	RYKSGPFHSAWYGMYVRC	169
	PdLEA2.7	RVKLGSLKLWKVTSRVRC	208

####  Phylogenetic analysis and multiple alignment

Global multiple sequence alignment of the PdLEA2 protein sequences produced low sequence identity ranging between 8.28–26.41%, indicating poor overall sequence conservation characteristic of the LEA2 superfamily. However, these sequence differences do not restrict it from producing a similar three-dimensional fold as discussed earlier. A search of all date palm protein sequences in NCBI RefSeq protein database, harboring a member of the LEA superfamily sequence, showed that most of these sequences have neither been well characterized, nor annotated appropriately. Needless, a phylogenetic tree was generated using PdLEA2 sequences (Fig. S1). PdLEA2.2 was identified to be close to NDR1/HIN1-like protein 6, PdLEA2.3 close to Lea14-A protein, PdLEA2.4 close to NDR1/HIN1-like protein 10, and PdLEA2.7 close to NDR1/HIN1-like protein 1, while PdLEA2.6 was located to proteins annotated as uncharacterized.

### Expression pattern of PdLEA2 genes under salt stress in two contrasting genotypes of date palm

**Figure 2:**
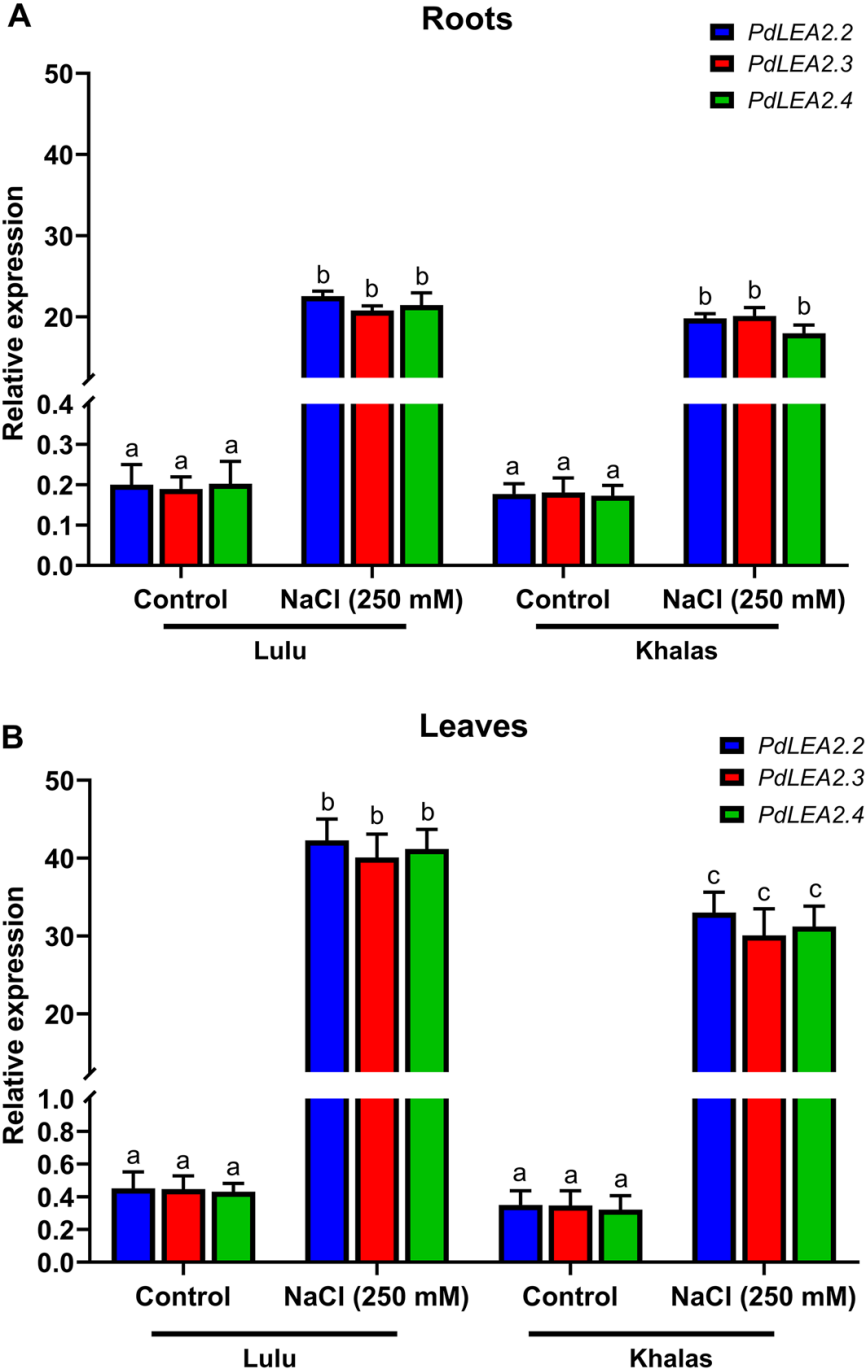
Expression profile of *PdLEA2* genes from the Lulu and Khalas cultivars under salt stress. (**A**) Roots (**B**) Leaves. The data of expression level are the means ± SD (n = 3) from the roots and leaves, analyzed with one-way ANOVA and Tukey’s HSD test. Different letters indicate a significant difference in the expression levels (*p* < 0.05).

The samples of RNA from roots and leaves of two contrasting genotypes grown under control and salt stress conditions were used for the Real-time PCR. In either, the salt sensitive date palm variety, Khalas, or the tolerant date palm variety, Lulu, the expression of the *PdLEA2.2*, *PdLEA2.3* and *PdLEA2.4* genes under salt stress, displayed a significant increase (*p* < 0.05) in both roots and leaves, compared to the control non-stressed plants (Fig. 2A-B). However, in roots under control or salinity stress condition, *PdLEA2* genes expression showed no significant difference between the tolerant and the sensitive varieties (Fig. 2A). In contrast, in leaves under salinity stress, a significant difference was observed between the *PdLEA2* genes expression level between the tolerant and the sensitive varieties, but not under the control condition (Fig. 2B). The *PdLEA2* genes level of expression were higher in leaves than in roots for both genotypes.

### Production and purification of recombinant PdLEA2 proteins

**Figure 3:**
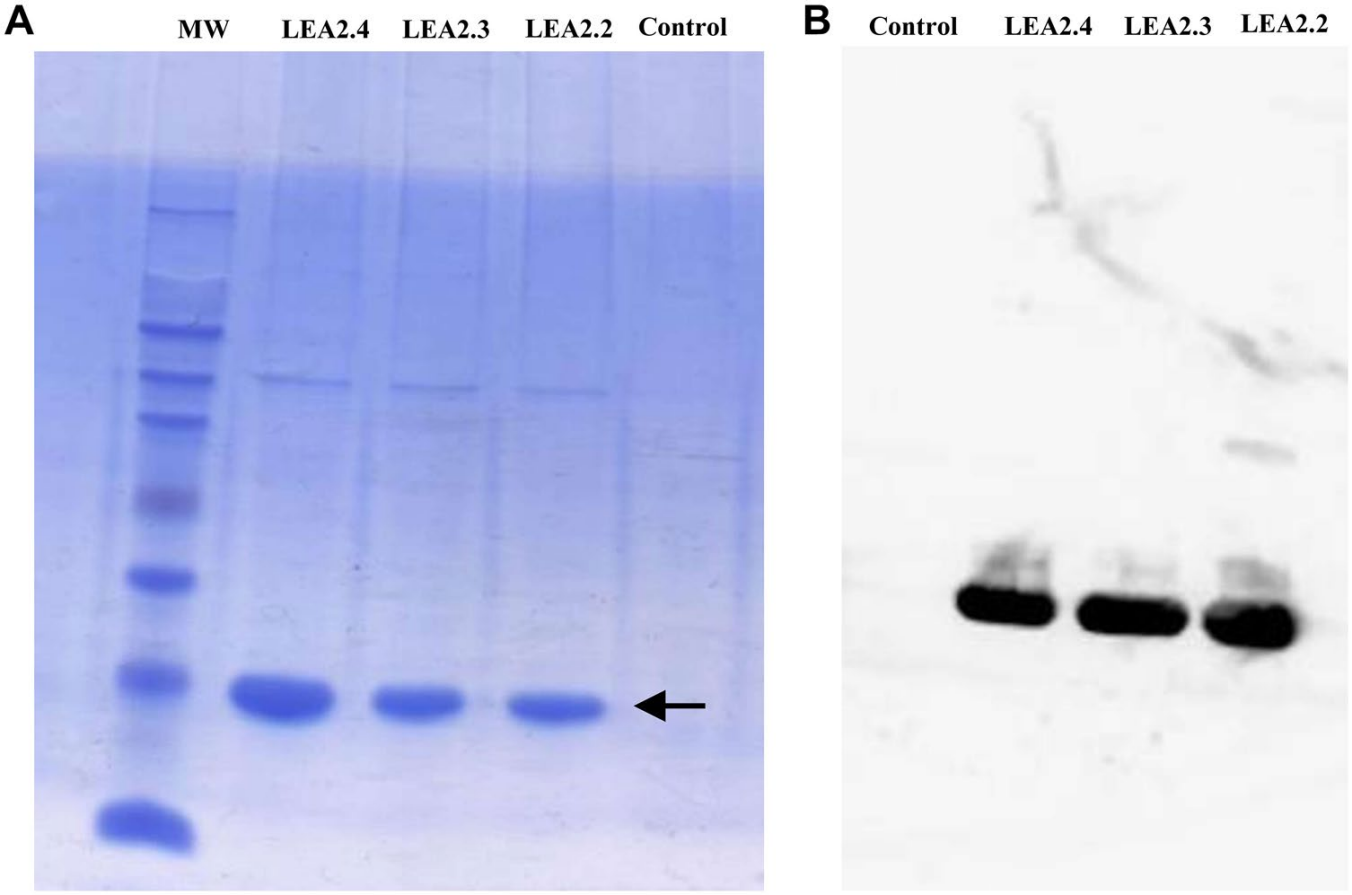
SDS-PAGE and Western blot analysis of PdLEA2 proteins expression and purification. (**A**) SDS-PAGE profile of recombinant proteins, Lane 1 is the protein ladder, Lanes 2, 3 and 4 are induced samples of PdLEA2.4, PdLEA2.3, and PdLEA2.2, and Lane 5 is the control. (**B**) Western blot of 1-Control, 2-PdLEA2.4, 3-PdLEA2.3, and 4-PdLEA2.2 proteins, identified using a His6-tag specific antibody.

The *PdLEA2.2*, *PdLEA2.3*, and *PdLEA2.4* ORFs were cloned in frame with the polyhistidine tag of the pET28a expression vector. Recombinant PdLEA2 proteins were expressed in *E. coli* cells (BL21 strain) and assessed by SDS-PAGE. After the induction with IPTG, PdLEA2 proteins accumulated in high amounts in the *E. coli* cells (Fig. 3A; Fig. S2). The affinity chromatography with nickel column was used to purify the overexpressed PdLEA2 proteins. The purity of PdLEA2 proteins was verified through Western blot analysis using an anti-His6 antibody (Fig. 3B; Fig. S2). As expected, the immunoblot revealed a band for the PdLEA2 proteins, but not with the control.

### PdLEA2 proteins stabilization of the LDH enzyme under heat stress conditions

**Figure 4:**
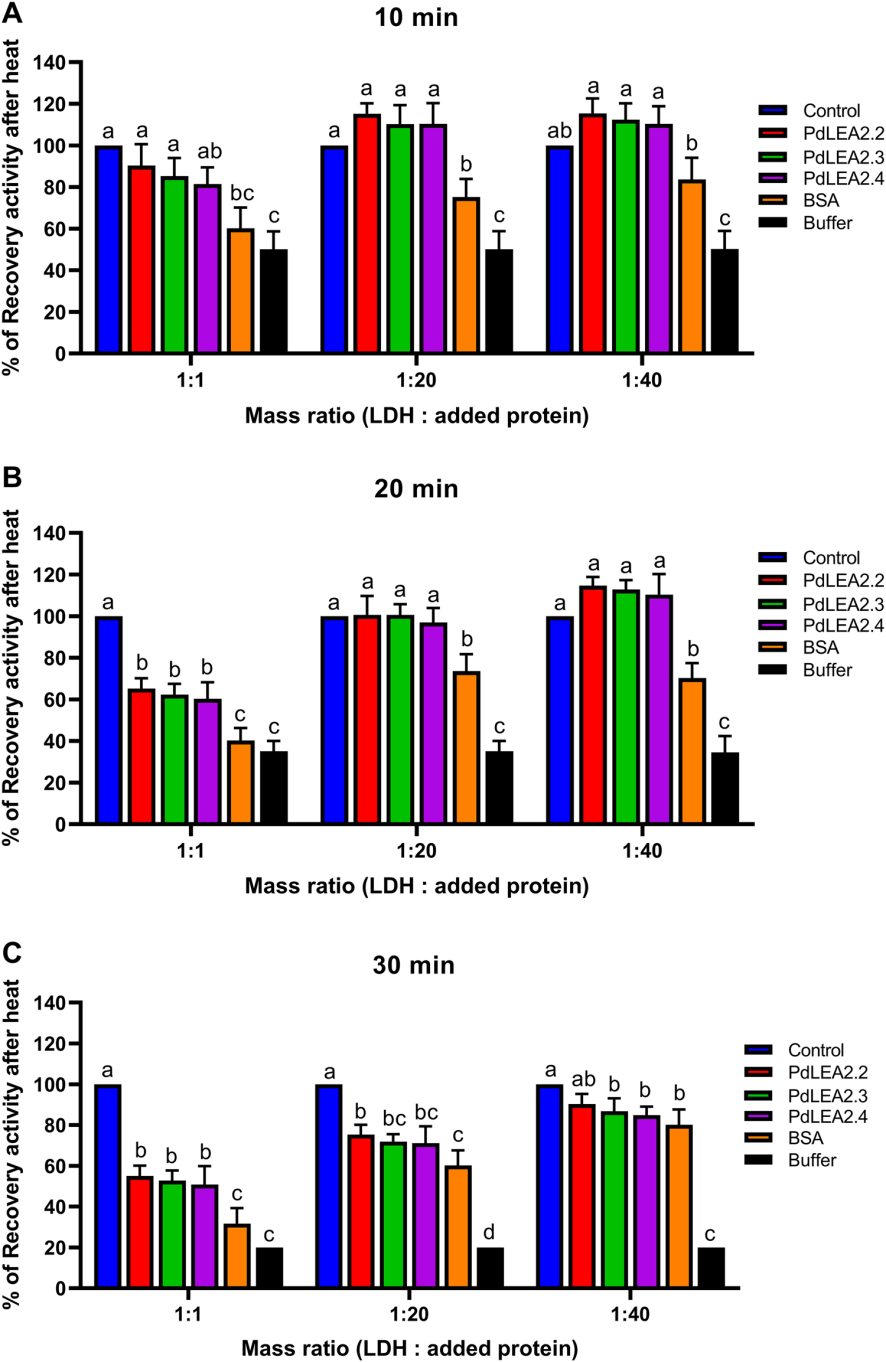
PdLEA2 protects LDH against inactivation and prevents its aggregation under heat stress in contrast to BSA and buffer. LDH enzyme activity recovery during heating at 50 °C for (**A**) 10 min (**B**) 20 min and (**C**) 30 min. Data are expressed as the means ± SD (n = 3), analyzed using one-way ANOVA. Different letters indicate a significant difference (*p* < 0.05) evaluated using Tukey’s HSD test.

The ability of PdLEA2.2, PdLEA2.3 and PdLEA2.4 to inhibit the LDH activity loss after heat stress was tested. The effects of PdLEA2.2, PdLEA2.3 and PdLEA2.4 proteins were compared with the BSA, a non-specific protectant, and with buffer treated LDH enzyme without adding protein (Fig. 4A-C). After 10 min of heat stress, at the mass ratios of 1:1, 1:20, and 1:40, a significant difference (*p* < 0.001) was observed in the stabilization of LDH enzyme with the PdLEA2 proteins, compared to BSA and buffer without additional protein. It was found that PdLEA2 proteins provided a greater shield to LDH than BSA and buffer, with the highest protection observed for the PdLEA2.2, 90% at 1:1 and 115% at 1:40 within 10 min of the heat stress (Fig. 4A). It was observed that half of LDH enzyme activity was lost with buffer following 10 min of heating at 50 °C. Similarly, after 20 min of heat stress, there was also a significant difference (*p* < 0.001) between the LDH enzymatic activity with PdLEA2 proteins, compared to BSA, and the buffer without addition of proteins at the three mass ratios (Fig. 4B). The percentage of enzymatic activity recovery was the highest for the PdLEA2.2 protein that augmented with increasing mass ratio of proteins, 65% (1:1) and 115% (1:40). Furthermore, a significant difference (*p* < 0.001) was observed within 30 min of enzyme activity under heat stress (Fig. 4C) between PdLEA2 proteins recovery activity, BSA and buffer without proteins at mass ratio of 1:1 and 1:20. However, at the mass ratio of 1:40 and after 30 min of heat stress, there was a significant difference (*p* < 0.001) between the PdLEA2 proteins and buffer without proteins, but no significant difference was observed between the stabilization provided by the PdLEA2 proteins and BSA. At the highest mass ratio (1:40) and after 30 min at 50 °C, PdLEA2.2 protected at least 90% of the enzymatic activity, while PdLEA2.3 and PdLEA2.4 preserved 86% and 84% of the enzyme activity, respectively. Whereas the recovery of enzyme activity was reduced to 80% for the BSA and to 20% for the buffer without protein. This indicated that the LEA2 proteins provided the stabilization of enzyme activity with no effects of the presence of a second nonspecific protein at the different mass ratios with longer incubation times. Thus, it was observed that the enzymatic activity of LDH was completely protected after 10 min, 20 min or 30 min of heat stress condition with the presence of PdLEA2 proteins at the mass ratios of 1:1, 1:20, and 1:40.

### PdLEA2 proteins inhibition of LDH aggregation under heat stress treatments

**Figure 5:**
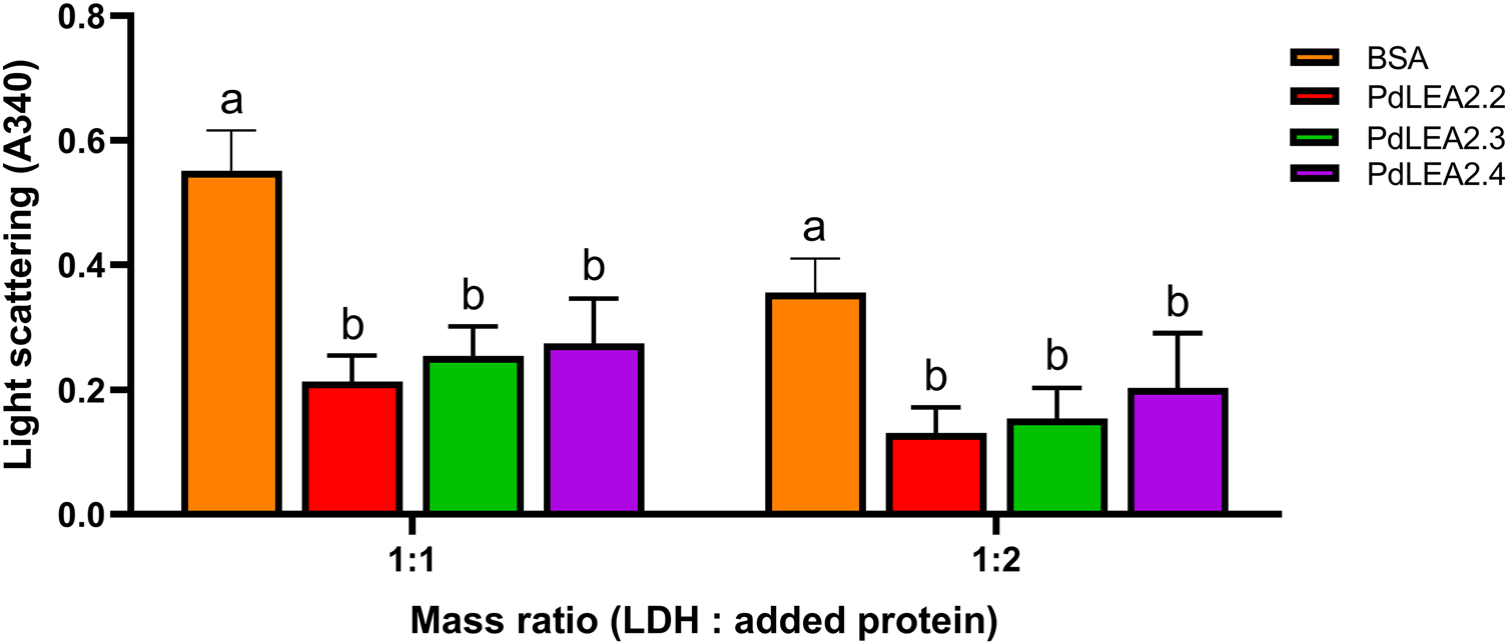
LDH anti-aggregation activity of PdLEA2 under heating stress. Enzymatic aggregation was monitored in spectrophotometer at the absorbance rate of 340 nm. PdLEA2 proteins and BSA were added to the LDH enzymatic reaction at two different mass ratios. Data are the means ± SD (n = 3), with different letters indicating a significant difference (*p* < 0.05) analyzed using Tukey’s HSD test after one-way ANOVA.

LDH enzyme forms aggregate when exposed to dehydration, heating, or freeze–thaw treatments. This study examined the capability of PdLEA2 proteins to decrease the aggregation of LDH enzyme under heat stress conditions through measuring the apparent light scattering absorbance of proteins solutions. The impact of PdLEA2 proteins on the enzymes was studied at two mass ratios of 1:1 and 1:2. It was observed that LDH formed massive aggregation after heating at 80 °C for 20 min. At both the mass ratios, there was a significant difference (*p* < 0.001) in the inhibition of LDH aggregate formation under the heat stress with addition of PdLEA2 proteins and BSA (Fig. 5). The existence of the PdLEA2 proteins declined the enzymatic aggregation of LDH at both the mass ratios in contrast to the BSA (Fig. 5). For enzymatic assay under heat stress, the absorbance in the presence of PdLEA2 was lowered more than the half of the aggregate formation in the presence of BSA in both the mass ratios (Fig. 5). It was found that LDH aggregation was consistently lower at a similar activity rate between the three PdLEA2 proteins.

### PdLEA2 improved the thermostability and activity of bglG

**Figure 6:**
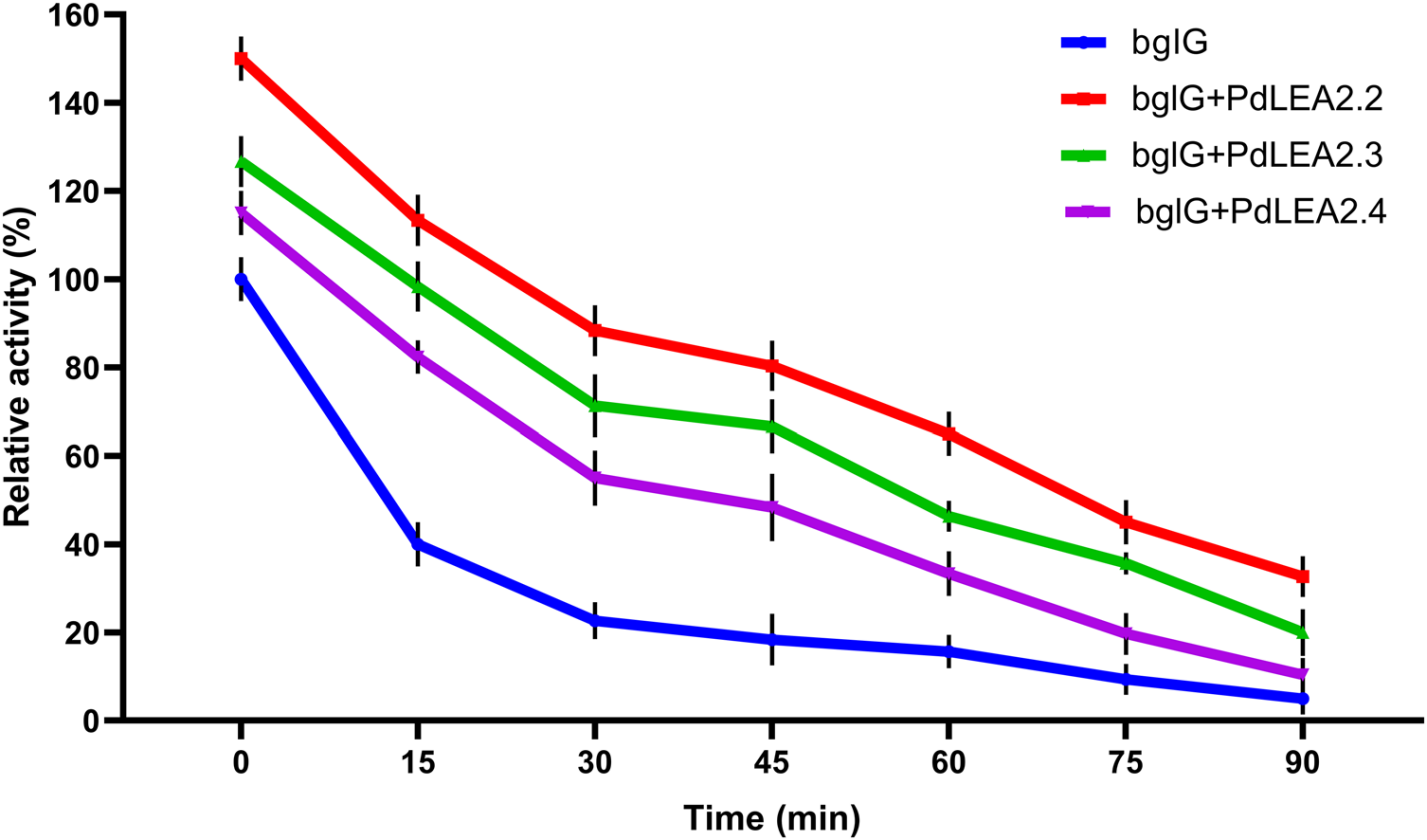
Effect of PdLEA2 proteins on the bglG enzymatic thermostability. bglG enzyme was incubated with and without PdLEA2 proteins at 70 °C during which the enzyme loses its catalytic activity. The relative activity was determined by taking aliquots at different time intervals. The data are the means ± SD (n = 3).

To test the protective effect of PdLEA2 proteins on bglG enzymatic activity at 70 °C, recombinant PdLEA2.2, PdLEA2.3 and PdLEA2.4 proteins (0.5 µg ml^−1^) were added to the reaction of the bglG enzyme. The treatments were performed with and without PdLEA2 proteins for 15 min time intervals up to 90 min. A significant difference (*p* < 0.001) was observed in the enzymatic activity with and without PdLEA2 proteins at the different time intervals of heat stress reaction (Fig. 6). The bglG enzyme activity decreased drastically in the absence of PdLEA2 proteins within 30 min to 22%, while it was found that PdLEA2.2 protein preserved 90% of the enzyme activity at the same time interval. In contrast, PdLEA2.3 and PdLEA2.4 proteins conserved 71% and 55% of the enzyme activity after 30 min of heat stress, respectively. Furthermore after 60 min of incubation, 65% of the enzyme relative activity was stabilized by PdLEA2.2, 46% and 33% by PdLEA2.3 and PdLEA2.4. Whereas it declined to 15% without the PdLEA2 proteins at 60 min of heat stress. After 90 min of heat stress, it was observed that the bglG enzyme activity was reduced to 5% in the absence of PdLEA2 proteins, and in the presence of PdLEA2.2, PdLEA2.3, and PdLEA2.4, the bglG enzyme activity was preserved to 33%, 20%, and 10%, respectively. This finding indicates that PdLEA2 proteins have a preservative effect on bglG under heating stress, providing an enhanced activity of the enzyme at higher temperatures in contrast to using bglG alone.

## Discussion

In plants, LEA2 proteins have been characterized functionally and are involved in responses to the environmental stresses of various plants, specifically drought-tolerant plants^23^, halophytes^24^, resurrection plants^15^, and cold tolerant plants^25^. In this study, LEA2 proteins from *P. dactylifera* were identified, characterized, and explored for the first time for their potential implication on enzyme stability and thermotolerance.

The *LEA2* genes are abundantly present in the *P. dactylifera* genome assembly, having sixty-two members, in comparison to fifty-two and forty-six in rice and sorghum, respectively^17^. In addition, there were thirty *LEA2* genes in *Prunus salicina*^26^, twenty-seven in *Solanum lycopersicum*^27^, fifty-three in populus^28^, twenty-nine in *Solanum tuberosum*^29^ and thirty-two in tea plant^4^. In contrast, the number of *LEA2* genes was much higher in *Ramonda serbica*^20^ and *Arachis hypogaea*^30^, which had 127 and 78 members, respectively. The abundance of *LEA2* genes in date palm can be depicted as being the last members to evolve among the *LEA* genes group or because of the whole gene duplication event within this group as it occurred in cotton plants^9^. The high abundance or redundancy of a particular gene group indicates the major part it plays in improving the plants survival. Date palm growing in the arid regions continuously faces harsh environmental conditions. Their growth under the environmental stresses can be attributed to the accumulation of diverse stress tolerant *LEA2* genes or due to the integration of these genes with several other gene regulatory mechanisms for activating adaptive responses.

The location of a gene on the chromosome plays an essential role in shaping an organism’s trait and its evolution. The *PdLEA2* genes were broadly distributed and located on different chromosomes in the date palm genome. The range of amino acid sequence of the identified PdLEA2 proteins were between 202 to 317 aa, which is larger than found in bay beans^31^ and tea plants^32^, but is in close range to *Rhododendron catawbiense* DHNs, RcDhn 1–5^33^. However, the molecular weight of PdLEA2 proteins were smaller than the *A. thaliana*^34^ and cotton9, which were extending between 67.2 to 160.7 kDa, respectively. The pI values of PdLEA2 proteins ranged between 4.85 to 9.96, indicating that the isolated PdLEA2 proteins were basic in nature, similar to *Brachypodium distachyon* LEA2 proteins^35^ and wheat LEA proteins^36^. The average pI of the PdLEA2 proteins displayed higher association with the *G. hirsutum* LEA groups, specifically DHNs and SMPs9.

In terms of hydrophobicity, GRAVY values indicated that two of the proteins, PdLEA2.3 and PdLEA2.4, were moderately hydrophilic, while PdLEA2.7 was hydrophobic and PdLEA2.2 and PdLEA2.6 were in between. Notably, Kyte-Doolittle plots indicated the presence of a long stretch of hydrophobic region which was predicted to form a transmembrane helix. The moderate hydrophilic nature of PdLEA2.3 and PdLEA2.4 proteins can enable them to form a hydrogen bond with water molecules, thereby get dissolved in water, which is a thermodynamically favored interaction. The hydrophilic property was previously observed in LEA2 proteins of other plants^37^. It allows them to get totally or partially disordered, a unique feature of the LEA proteins. The hydrophilic property is further required to form flexible structural elements, such as molecular chaperones, that are essential for the plant’s protection against desiccation^38^. On the other hand, the hydrophobic nature of the PdLEA2.7 proteins enables them to fold spontaneously into complex structures and further allow the discharge of nonpolar amino acids from the solvents. This attribute commonly occurs in the water channel proteins such as aquaporins (AQPs), which are highly hydrophobic and play a significant part in drought and salinity stress tolerance of plants^39^. Instability index showed that most of the isolated PdLEA2 proteins were unstable in contrast to that of *Sorghum bicolor* LEA, SbLEA and *S. lycopersicum* LEA, SiLEAs that were similar to stable PdLEA2.3 protein^27,40^. PdLEA2 proteins displayed high aliphatic index suggesting their relative volume is occupied largely by aliphatic side chains such as alanine, isoleucine, leucine, and valine that enhances their thermostability. LEA proteins are not transmembrane proteins as they can be located in nucleus, chloroplast, mitochondria, and cytoplasm^41^. Contrarily, PdLEA2 proteins exhibited transmembrane helices, which indicates their expression in subcellular compartments. The presence of at least one transmembrane α-helix was similarly identified in *R. serbica* LEA2 proteins20.

The conserved domain analysis of PdLEA2 proteins revealed the presence of a LEA_2 superfamily domain, pfam03168, in PdLEA2.2, PdLEA2.4, and PdLEA2.7. This LEA_2 domain-containing proteins have been correlated with various plant tolerance responses against several abiotic stresses such as heat, drought, salinity, osmotic stress, UV damage, and oxidative stress^42–45^. Furthermore, PdLEA2.3 consisted of WHy domain and LEA_2 superfamily, smart00769. The presence of WHy domain sequence has been found as an ORF in certain bacterial genomes of phylum Firmicutes^46^. It has been reported that the recombinant bacterial WHy protein exhibited stress tolerance phenotype in *E. coli* and provided protection to protein denaturation *in vitro*^46^. Protein denaturation implies the damage of the tertiary structure, resulting in the loss of stability and structure of the protein. Moreover, the PdLEA2.4 and PdLEA2.6 had the LEA_2 superfamily domain, cl12118, which was also identified in cotton LEA2 proteins^9^.

The majority of LEA2 proteins have typical motifs that are essential for their identification. In PdLEA2 proteins, four distinctive motifs were found to be present. The motif length varied between 8 and 24 amino acids. The motif 2 was occurring in all the PdLEA2 proteins, whereas the motif 1 was present in PdLEA2.2 and PdLEA2.4 proteins only. Similarly, in PdLEA2.2, PdLEA2.4, and PdLEA2.6 only conserved motif 3 was present. In addition, motif 4 was found in PdLEA2.2, PdLEA2.4 and PdLEA2.7. The presence of a common motif composition indicates identical functional specificity within the PdLEA2 proteins subgroup as identified in *S. tuberosum*^47^. Identical results of LEA proteins group-specific conserved motifs were reported earlier for Arabidopsis^34^, *S. lycopersicum*^27^, Prunus^26^ , poplar28, maize^48^, Brassica^49^, and cotton^9^.

The LEA2 proteins are predicted to be IDPs^6^. The secondary and 3D structural analysis of all five PdLEA2 proteins displayed a disordered region at the N-terminal site. This region was followed by an α-helix and a tertiary structure consisting of three β-hairpins and β-strands. Thus, the most predominant folded secondary structure was the β-strand, with random coils distributed throughout PdLEA2 protein sequences. The structure of PdLEA2 proteins were similar to the secondary structure prediction in bay bean and *R. serbica* LEA proteins in relation to β-sheet, α-helix, and random coil content^31^. However, this is in contradiction with the wheat LEA proteins that had lower β-sheet strands^36^.

Due to the absence of validation and good annotation for LEA2 proteins, the phylogenetic tree generated using date palm protein sequences harboring members of the LEA2 superfamily showed that, in most cases, PdLEA2 proteins were located next to proteins annotated as NDR1/HIN1-like in the date palm genome. NDR1/HIN1-like (NHL) gene groups include the Harpin-induced gene 1 (HIN1) and Nonrace-specific disease resistance gene 1 (NDR1)^50^. HIN1 gets accumulated by a harpin protein that is involved in different plant defense responses to abiotic stresses50. Whereas the cloning of NDR1 gene from *A. thaliana* indicated its responsive function to plant disease resistance^51^.

*LEA2* genes are largely expressed in response to abiotic stress in plants^6^. In this study, *PdLEA2* genes displayed a relatively higher expression in leaves of the tolerant and sensitive varieties, Lulu and khalas seedlings after salinity stress treatments, respectively. This finding is consistent with the expression of LEA genes in cotton, *S. bicolor*, and maize plants vegetative tissues^9,40,48^. Leaves are the major plant parts that are inflicted by the salinity stress. The increased accumulation of *PdLEA2* transcripts in leaf tissues indicates their role in preserving the structural integrity and preventing the salinity damage to the membranes. Furthermore, leaves are major site for photosynthesis, which get affected due to the excess release of reactive oxygen species (ROS) under salt stress^52^. ROS are detrimental to plant growth and high *PdLEA2* genes expression in the leaves, indicate their involvement in the scavenging of the ROS through the enhancement of the activities of antioxidant enzymes, similar to *Oryza sativa LEA5* gene^53^.

Protein denaturation is a frequent physiological phenomenon occurring in plant cells faced with abiotic stress. Different factors affect the enzymatic activities such as the temperature, pH, substrate and enzyme concentrations, and the presence or absence of inhibitors and activators in the reaction. Thermal stabilization of enzyme activity such as Glucose oxidase (GOD) has been reported in the presence of trehalose, a most widely known compatible solute acting as a chemical chaperone^54^. In the present study, it was found that PdLEA2 proteins protected the LDH and bglG enzymatic activities from the damages of heating stress during their reaction conditions. The presence of PdLEA2 proteins allowed higher LDH and bglG enzymatic activities recovery under heat stress, indicating that the isolated PdLEA2 proteins have strong ability to protect the enzymes. The enzymatic protection of the LEA2 proteins was more efficient than provided by BSA. This can be attributed to the disorder structure, transmembrane helix, and folded region consisting of β-sheets of PdLEA2 proteins during the heat stress or due to their ability to act as chaperones and bind to membranes, metal ions and water. The LDH and bglG loss of catalytic activity was not only preserved, but PdLEA2 proteins further prevented the aggregation of LDH enzyme. Particularly during drying, decrease in aggregation, has been found for other LEA proteins from plants, which was associated to the hypothesis of molecular shield^55^. Several studies have indicated that DHNs can protect LDH and bglG enzyme activities against the damage caused by various stresses. In relation to PdLEA2 proteins, it was found that CdDHN4-L and CdDHN4-S recovered the LDH enzymatic activities during free-thaw damage and heating stress^56^. In addition, it was reported in the presence of *Picea Wilsonii* DHN, PicW1, LDH enzyme activity was higher than the blank control and BSA at 43 °C to 55°C^57^. Alternatively, the conditions of heating and freezing stress causes water stress and certain DHNs are found to play the role of antiaggregant agents of the other protein or enzyme molecules under this stress^58^. The function of PdLEA2 proteins in protecting enzyme activities during heat stress was similar to the wheat LEA3 protein, TdLEA355. Indeed, the addition of TdLEA3 at the highest mass ratio in the reaction, preserved 90% of the LDH enzymatic activity at 48 °C after 30 min. The structural domains of LEA2 proteins and the mechanism controlling the enzymatic activity protection under stress conditions, has been investigated in few studies as a heat protection mechanism of LEA2 proteins. In *Brassica napus*, LEA3 proteins protected LDH under desiccation stress, which was attributed to its hydrophilic nature, β-sheets, α-helix, and random coil propensity^38^. In the present study, PdLEA2.2, PdLEA2.3 and PdLEA2.4 were moderately hydrophilic, had β-sheets and random coil propensity, which may have contributed to the protection of LDH enzyme under the heat stress. Moreover, in cryoprotection assay, formation of random coil occurred in Arabidopsis COR15^59^, which indicated the role of random coil structure in the enzymes cryoprotection. Few studies have been conducted in investigating the role of LEA2 proteins on protecting the bglG enzyme under heat stress. In our previous study, we found that DHN-5 from wheat played a relevant role in protecting the enzyme bglG against heat stress^60^. Truncation assay of DHN-5 indicated that K-segments were vital to the thermal protection of LDH and bglG^61^. It was observed that the truncated forms of DHN-5 that contained only one or two K-segments were able to protect LDH and bglG enzymatic activities against the damages caused by various stresses in vitro albeit to lesser extent than the wild-type protein. Beside the impact of K-segments in improving the LDH and bglG heat stability, these truncated forms properly refolded the enzymes after heat stress as the wild-type protein. However, the PdLEA2 proteins are lacking any K-segment and protected the bglG enzyme activity, indicating a possible role of hydrophilicity and β-sheets structure in protecting the bglG enzyme under heat stress.

## Conclusion

In this study, LEA2 proteins from date palm were characterized, and the functional analysis elucidated their roles in protecting enzymes. The PdLEA2 proteins had high sequence similarity, hydrophilicity, and were disordered in structure. It was found that the isolated PdLEA2 proteins from date palm protected the enzymatic stability and activity of LDH and bglG enzymes under heat stress. The protective role of PdLEA2 proteins can be due to their disorder structure, transmembrane helix, and folded region consisting of β-sheets, which enables them to stabilize various partner molecules such as enzymes or target proteins in different cellular compartments during the heat stress. The opulence of *LEA2* genes in date palm and their functional characterization sets a major foundation for further research in understanding the evolutionary relation of *LEA2* gene family and their potential role in plants tolerance to abiotic stress conditions. In addition, the generation of transgenic plants overexpressing *PdLEA2* genes will provide protection against environmental constraints such as salinity, drought, and heat stresses. Moreover, the use of fermentation for the outgrowth of bacterial cells at a large volume to enhance the production of recombinant PdLEA2 proteins is considered as a potential application from this study. The present findings reinforce the proposition that PdLEA2 proteins are vital molecules that can be harnessed as molecular chaperones for developing novel recombinant thermoresistant enzymes with improved rate of reaction and specificity.

## Methods

Our experimental research carried out on cultivated date palm from tissue culture was conducted in conformity with all applicable agricultural and genetic resource laws for the management of plants. Permission was obtained to collect the plant materials and experimental methods were performed in accordance with the relevant guidelines.

### Plant material and stress treatment

Date palm seedlings from the tissue cultured Lulu and Khalas cultivars were grown in ½ MS liquid medium and maintained in growth chamber with a temperature of 25 ± 2 °C, a photoperiod of 16 h light and 8 h of dark at 280 μmol photons m^−2^ s^−1^, and with a relative humidity of 70%. For salt treatment, the seedlings received 250 mM NaCl in the ½ MS liquid medium, while the control seedlings received sterile water. The *P. dacrylifera* seedlings were raised under the same ambient conditions in the growth chamber for two additional weeks. Thereafter, the roots were collected from the control and the salt treated plants, then stored at -80 °C for downstream analysis.

### Identification of LEA2 gene sequences from date palm

The *P. dactylifera* genome and the root transcriptome data from the cultivar Khalas were used to identify the *LEA2* gene sequences. All the identified candidates were analyzed using the Pfam database (http://pfam.sanger.ac.uk) to confirm conserved domains. Five *PdLEA2* genes (*PdLEA2.2*, *PdLEA2.3*, *PdLEA2.4*, *PdLEA2.6*, and *PdLEA2.7*) were selected and specific primers were designed for cloning the respective *LEA2* genes (Table S2).

### Molecular cloning of P. dactylifera LEA2 genes

The total RNA was isolated from the seedling roots of control and salt stressed *P. dactylifera*, Khalas variety following the manufacturer’s instructions using the RNeasy® Plant Mini Kit (QIAGEN, Germany). The PrimeScript RT-PCR Kit (TaKaRa Bio, Japan) was used to synthesize the first strand cDNA from the isolated RNAs. The synthesized cDNAs served as the templates for PCR with specific primers designed with Primer Premier 6.0. The PCR reaction was performed for 30 cycles with an initial denaturation of 3 min at 95 °C, followed by a denaturation of 30 s for 95 °C, annealing for 30 s at 55 °C, extension for 1 min at 72 °C and with a final extension of 7 min at 72 °C. The PCR products were cut off from the agarose gel and purified with spin columns of GenElute minus ethidium bromide (Sigma-Aldrich). The purified products were cloned into the pMiniT 2.0 vector using the NEB PCR Cloning Kit (New England, BioLabs, Inc.) and sequenced by outsourcing with Macrogen TM (South Korea) in both directions to verify the sequence of the *LEA2* genes.

### Expression analysis of PdLEA2.2, PdLEA2.3, and PdLEA2.4 genes in different date palm tissues

Total RNA was isolated from leaves and roots of control and salt stressed *P. dactylifera* seedlings of two contrasting varieties, Khalas and Lulu, sensitive and tolerant, respectively using the RNeasy® Plant Mini Kit (QIAGEN, Germany). One μg of total RNA from each sample was processed with RNase-free DNase I and reverse transcribed into cDNA in accordance with the manufacturer’s instruction of SuperScript III Reverse transcriptase kit from Invitrogen. Using the Applied Biosystems machine of Real-Time PCR, quantitative real-time PCR was conducted with SYBRTM Select Master Mix in 96-well plates (Applied Biosystems). The PCRs were conducted in a 10 µl terminal volume that contained 3 µl cDNA (attained from 40 ng of DNase-treated RNA), 0.5 µl (at 10 µM) of primers, 5 µl of 2 × SYBR Green I master mix, and 1 µl of RNase-free water (Sigma). The process involved a preliminary denaturation for 10 min at 94 °C, then 45 cycles with 94 °C for 10 s, 59 °C for10 s, 72 °C for 15 s, followed by a liquification curve with 5 s at 95 °C, 1 min at 65 °C, and 5 min with temperature increasing from 65 °C to 97 °C. The Primer3 Input software was used to create the primers for real time PCR that are shown in Table S3. The formula: 2^-DDCT^, where DDCT resembles to [(CT of Target gene—CT of Actin gene) under salt stress condition— (CT of Target gene—CT of Actin gene) under normal condition], was used to calculate the relative expression level.

### Expression, production, and purification of recombinant PdLEA2.2, PdLEA2.3, and PdLEA2.4 proteins

Out of the five isolated *LEA2* genes from date palm, three were selected for protein expression in *E. coli*, based on their hydropathy index (moderately hydrophilic). Accordingly, the full-length ORF of *PdLEA2.2*, *PdLEA2.3*, and *PdLEA2.4* (GenBank accessions: OQ348181, OQ348184, OQ348183) were amplified from the date palm Khalas cDNA with Phusion high fidelity DNA polymerase (New England, Biolabs), using specific primers with added restriction sites BamHI and EcoRI at the 5′- and 3′- ends (Table S4). The *PdLEA2.2*, *PdLEA2.3*, and *PdLEA2.4* ORFs were cloned into the BamHI and EcoRI sites of the pET28a, expression vector of *E. coli*. Recombinant proteins expression of *PdLEA2* in *E. coli* BL21 (DE3) were induced at 37 °C with the addition of 1 mM isopropylthio-β-galactoside (IPTG). The cultivation of *E. coli* cells was performed for an additional 4 h, then harvested and lysed with NENT buffer (100 mM NaCl, 1 mM EDTA, 0.5% NP40, 20 mM Tris–HCl, and pH at 7.9) containing the protease inhibitor phenylmethylsulfonyl fluoride (PMSF). Protein purification was performed by using the HisLink Protein Purification Resin according to the manufacturer instructions (Promega). By using SDS-PAGE, five μg of protein from each sample were separated. Protein bands were analyzed by Coomassie blue staining and identified by Western blot using a His6-tag specific antibody.

### LDH and bglG protection assays

The bovine heart LDH was obtained from Sigma–Aldrich and diluted using 10 mM Na_3_PO_4_ at pH 7.4 according to the instructions of the manufacturer. One μl of 20 μg/μl LDH was transferred to a buffer that contained 20 μl of PdLEA2 or BSA at three different mass ratios (LDH: LEA/BSA), which were 1:1, 1:20, and 1:40. Vacuum-drying of the samples was performed in a Speed Vac to 6 μl of final volume that were rehydrated with the introduction of 14 μl of buffer. The impact of heating was tested with the sample’s treatment to 50 °C for up to 60 min. The enzyme activity was determined by adding one ml of freshly assembled assay buffer (at pH 7.4 of 10 mM Na_3_PO_4_, with 2 mM NADH, and 10 mM pyruvic acid) into the samples of LDH enzyme. At 340 nm, NADH oxidation was observed for 3 min, during which a linear rate of reaction was present. The enzyme activity was calculated using the rate of absorbance decrease (ΔOD/min) × 8095 = U l^−1^. The assay samples were analyzed in triplicates.

The effect of PdLEA2 proteins on the thermostability of the bglG enzyme was tested by using purified PdLEA2 proteins at optimal concentration of 0.5 µg ml^−1^ as additive in the bglG assay for two different temperatures, 50 °C as the optimal temperature and 70 °C during which its catalytic efficiency is lost drastically. The enzyme bglG from *Aspergillus niger* was obtained from Megazyme and used according to the manufacturer’s instructions. Thermal stability of bglG was measured through the incubation of the purified enzyme at the required temperatures for 30 min intervals, using 1 mM of para-nitrophenyl β-D-glucopyranoside as substrate, followed by measuring the relative activity. The reaction was stopped with the addition of 0.6 ml of 0.4 M Glycine–NaOH buffer (pH 10.8), and the released p-nitrophenol was evaluated at 400 nm. 18,000 M^−1^ cm^−1^ was used as the molecular extinction coefficient of p-nitrophenol. One unit of enzymatic activity was determined as the quantity of bglG needed to liberate one mol of p-nitrophenol under the assay condition per min.

### Bioinformatics analysis of PdLEA2 sequences and structure

Various physical and chemical parameters of the PdLEA2 proteins, including molecular mass, theoretical pI, instability index, aliphatic index, and grand average of hydropathy (GRAVY), were assessed using Exapsy ProtParam^62^. Hydropathy analysis of the PdLEA2 protein sequences was performed using Expasy ProtScale based on the Kyte & Doolittle scale62. Disordered regions in the protein sequences were analyzed using a CS-BLAST against the Database of Disordered Protein Predictions (D^2^P^2^)^63^, which aggregates results from several disorder prediction tools and DISOPRED^64^. Conserved domains were analyzed using NCBI’s CD-Search tool and protein motifs of PdLEA2 sequences were identified using Multiple EM for Motif Elicitation (MEME)^65^. Secondary structure prediction was performed using PSIPRED^66^ and transmembrane (TM) regions were predicted using MEMSTAT^67^, DeepTMHMM^68^ and TOPCONS^69^. Models of the three-dimensional structure of PdLEA2 sequences were generated using an in-house installation of AlphaFold2^70^.

To generate a phylogenetic tree of PdLEA2 proteins, all available *P. dactylifera* LEA2 protein sequences were obtained from NCBI RefSeq Protein database. The presence of LEA2 superfamily was confirmed in these sequences using NCBI’s CD-Search tool. The obtained and the PdLEA2 sequences reported here were aligned using MAFFT version 4.790^71^ in Geneious Prime 2022.2.2 (https://www.geneious.com). A phylogenetic tree was generated using EBI’s Simple Phylogeny tool using the neighbor joining method. The tree was visualized, annotated, and rendered using Interactive Tree of Life v5^72^.

### Statistical analysis

Statistical parameters such as mean and standard deviation (SD) were calculated for the PdLEA2 genes expression level and enzymatic activities of LDH and bglG under heat stress condition with different PdLEA2 protein treatments. Three biological replicates were used for each of the enzymatic assays and PdLEA2 expression analysis. Data were analyzed using one-way ANOVA with Tukey’s HSD test for the evaluation of significant differences between PdLEA2 proteins and control treatments under heat stress. Each of the variable’s normality and homoscedasticity were evaluated. The analyses were performed using the R statistical software.

## Supplementary Information

Supplementary Figures.

Supplementary Tables.

## Data Availability

The date palm LEA2 gene sequences are available at NCBI DataSets (https://www.ncbi.nlm.nih.gov) under the accession nos. OQ348181.1 (PdLEA2.2), OQ348184.1 (PdLEA2.3), OQ348183.1 (PdLEA2.4), OQ348182.1 (PdLEA2.6) and OQ348185.1 (PdLEA2.7). All other study data are included in the article and in additional information as supplementary files.

## References

[1] Hernández-Sánchez (2020). LEAfing through literature: Late embryogenesis abundant proteins coming of age-achievements and perspectives. J. Exp. Bot..

[2] Graether SP (2022). Proteins involved in plant dehydration protection: The late embryogenesis abundant family. Biomolecules.

[3] Dure L, Greenway SC, Galau GA (1981). Developmental biochemistry of cottonseed embryogenesis and germination: Changing messenger ribonucleic acid populations as shown by in vitro and in vivo protein synthesis. Biochemistry.

[4] Jin X (2019). Genome-wide identification and expression analyses of the LEA protein gene family in tea plant reveal their involvement in seed development and abiotic stress responses. Sci. Rep..

[5] Yang Z (2022). Characterization of a novel *TtLEA2* gene from Tritipyrum and its transformation in wheat to enhance salt tolerance. Front. Plant Sci..

[6] Abdul Aziz M, Sabeem M, Mullath SK, Brini F, Masmoudi K (2021). Plant group II LEA proteins: Intrinsically disordered structure for multiple functions in response to environmental stresses. Biomolecules.

[7] Hao Y, Hao M, Cui Y, Kong L, Wang H (2022). Genome-wide survey of the dehydrin genes in bread wheat (Triticum aestivum L.) and its relatives: Identification, evolution and expression profiling under various abiotic stresses. BMC Genom..

[8] Zhou M, Peng N, Yang C, Wang C (2022). The Poplar (*Populus trichocarpa*) dehydrin gene *PtrDHN-3* enhances tolerance to salt stress in Arabidopsis. Plants.

[9] Magwanga RO (2018). Characterization of the late embryogenesis abundant (LEA) proteins family and their role in drought stress tolerance in upland cotton. BMC Genet..

[10] Liu Y, Wang L, Zhang T, Yang X, Li D (2017). Functional characterization of KS-type dehydrin ZmDHN13 and its related conserved domains under oxidative stress. Sci. Rep..

[11] Bao F (2017). Overexpression of *Prunus mume* dehydrin genes in tobacco enhances tolerance to cold and drought. Front. Plant Sci..

[12] Narusaka Y (2003). Interaction between two cis-acting elements, ABRE and DRE, in ABA-dependent expression of Arabidopsis *rd29A* gene in response to dehydration and high-salinity stresses. Plant J..

[13] Solano R (1995). Dual DNA binding specificity of a petal epidermis-specific MYB transcription factor (MYB.Ph3) from *Petunia hybrida*. EMBO J..

[14] Simpson S (2003). Two different novel cis-acting elements of erd1, a clpA homologous Arabidopsis gene function in induction by dehydration stress and dark-induced senescence. Plant J..

[15] Giarola V, Challabathula D, Bartels D (2015). Quantification of expression of dehydrin isoforms in the desiccation tolerant plant *Craterostigma plantagineum* using specifically designed reference genes. Plant Sci..

[16] Müller H (2017). The desert plant *Phoenix dactylifera* closes stomata via nitrate-regulated SLAC1 anion channel. New Phytol..

[17] Al-Mssallem IS (2013). Genome sequence of the date palm *Phoenix dactylifera* L. Nat. Commun..

[18] Sabeem M (2022). Enhancing growth and salinity stress tolerance of date palm using *Piriformospora indica*. Front. Plant Sci..

[19] Hazzouri KM (2020). Prospects for the study and improvement of abiotic stress tolerance in date palms in the post-genomics era. Front. Plant Sci..

[20] Pantelić A, Stevanović S, Komić SM, Kilibarda N, Vidović M (2022). In silico characterisation of the late embryogenesis abundant (LEA) protein families and their role in desiccation tolerance in *Ramonda serbica* Panc. Int. J. Mol. Sci..

[21] Karagüler NG (2007). Protein engineering applications of industrially exploitable enzymes: *Geobacillus stearothermophilus* LDH and *Candida methylica* FDH. Biochem. Soc. Trans..

[22] Yin B (2019). Identification and molecular characterization of a psychrophilic GH1 β-glucosidase from the subtropical soil microorganism Exiguobacterium sp. GXG2. AMB Express.

[23] Chiappetta A (2015). A dehydrin gene isolated from feral olive enhances drought tolerance in Arabidopsis transgenic plants. Front. Plant Sci..

[24] Yamamoto N (2015). Comprehensive analysis of transcriptome response to salinity stress in the halophytic turf grass *Sporobolus virginicus*. Front. Plant Sci..

[25] Guo X, Zhang L, Zhu J, Liu H, Wang A (2017). Cloning and characterization of SiDHN, a novel dehydrin gene from Saussurea involucrate Kar. et Kir. that enhances cold and drought tolerance in tobacco. Plant Sci..

[26] Du D (2013). Genome-wide identification and analysis of late embryogenesis abundant (LEA) genes in *Prunus mume*. Mol. Biol. Rep..

[27] Cao J, Li X (2015). Identification and phylogenetic analysis of late embryogenesis abundant proteins family in tomato (*Solanum lycopersicum*). Planta.

[28] Lan T, Gao J, Zeng QY (2013). Genome-wide analysis of the LEA (late embryogenesis abundant) protein gene family in *Populus trichocarpa*. Tree Genet. Genomes.

[29] Charfeddine S, Saïdi MN, Charfeddine M, Gargouri-Bouzid R (2015). Genome-wide identification and expression profiling of the late embryogenesis abundant genes in potato with emphasis on dehydrins. Mol. Biol. Rep..

[30] Huang R (2022). Genome-wide identification, evolutionary and expression analyses of LEA gene family in peanut (Arachis hypogaea L.). BMC Plant Biol..

[31] Lin R (2021). Genome-wide analysis of the late embryogenesis abundant (LEA) and abscisic acid-, stress-, and ripening-induced (ASR) gene superfamily from *Canavalia rosea* and their roles in salinity/alkaline and drought tolerance. Int. J. Mol. Sci..

[32] Wang W (2019). The late embryogenesis abundant gene family in tea plant (*Camellia sinensis*): Genome-wide characterization and expression analysis in response to cold and dehydration stress. Plant Physiol. Biochem..

[33] Wei H (2019). Identification and characterization of five cold stress-related Rhododendron dehydrin genes: Spotlight on a FSK-type dehydrin with multiple F-segments. Front. Bioeng. Biotechnol..

[34] Hundertmark M, Hincha DK (2008). LEA (late embryogenesis abundant) proteins and their encoding genes in *Arabidopsis thaliana*. BMC Genom..

[35] Filiz E, Ozyigit II, Tombuloglu H, Koc I (2013). In silico comparative analysis of LEA (late embryogenesis abundant) proteins in *Brachypodium distachyon* L. Plant Omics.

[36] Bhattacharya S, Dhar S, Banerjee A, Ray S (2019). Structural, functional, and evolutionary analysis of late embryogenesis abundant proteins (LEA) in *Triticum aestivum*: A detailed molecular level biochemistry using in silico approach. Comput. Biol. Chem..

[37] Komić SM, Jovanović VS, Pantelić A, Vidović M (2022). Structural characterisation of late embryogenesis abundant proteins in *Ramonda serbica* Panč. Biologia Serbica.

[38] Liu Y (2019). Functional assessment of hydrophilic domains of late embryogenesis abundant proteins from distant organisms. Microb. Biotechnol..

[39] Sreedharan S, Shekhawat UKS, Ganapathi TR (2013). Transgenic banana plants overexpressing a native plasma membrane aquaporin MusaPIP1;2 display high tolerance levels to different abiotic stresses. Plant Biotechnol. J..

[40] Nagaraju M (2019). Genome-scale identification, classification, and tissue specific expression analysis of late embryogenesis abundant (LEA) genes under abiotic stress conditions in *Sorghum bicolor* L. PLoS ONE.

[41] Krogh A, Larsson B, Von Heijne G, Sonnhammer EL (2001). Predicting transmembrane protein topology with a Hidden Markov model: Application to complete genomes. J. Mol. Biol..

[42] Artur MAS, Zhao T, Ligterink W, Schranz E, Hilhorst HWM (2018). Dissecting the genomic diversification of late embryogenesis abundant (LEA) protein gene families in plants. Genome Biol. Evol..

[43] Battaglia M, Covarrubias A (2013). Late embryogenesis abundant (LEA) proteins in legumes. Front. Plant. Sci..

[44] He S (2012). Molecular characterization and functional analysis by heterologous expression in *E. coli* under diverse abiotic stresses for OsLEA5, the atypical hydrophobic LEA protein from *Oryza sativa* L. Mol. Gen. Genomics.

[45] Jia F (2014). Overexpression of Late Embryogenesis Abundant 14 enhances Arabidopsis salt stress tolerance. Biochem. Biophys. Res. Commun..

[46] Mertens J, Aliyu H, Cowan DA (2018). LEA Proteins and the evolution of the WHy domain. Appl. Environ. Microbiol..

[47] Chen Y (2019). The role of the late embryogenesis-abundant (LEA) protein family in development and the abiotic stress response: A comprehensive expression analysis of potato (*Solanum tuberosum*). Genes.

[48] Li X, Cao J (2016). Late Embryogenesis Abundant (LEA) gene family in maize: Identification, evolution, and expression profiles. Plant Mol. Biol. Rep..

[49] Liang Y (2016). Genome-wide identification, structural analysis and new insights into late embryogenesis abundant (LEA) gene family formation pattern in *Brassica napus*. Sci. Rep..

[50] Bao Y (2016). Overexpression of the NDR1/HIN1-Like gene NHL6 modifies seed germination in response to abscisic acid and abiotic stresses in Arabidopsis. PLoS ONE.

[51] Century KS (1997). NDR1, a pathogen-induced component required for Arabidopsis disease resistance. Science.

[52] Chen G, Zheng D, Feng N, Zhou H, Mu D, Zhao L, Shen X (2022). Physiological mechanisms of ABA-induced salinity tolerance in leaves and roots of rice. Sci. Rep..

[53] Huang L (2018). An atypical late embryogenesis abundant protein OsLEA5 plays a positive role in ABA-induced antioxidant defense in *Oryza sativa* L. Plant. Cell Physiol..

[54] Paz-Alfaro KJ, Ruiz-Granados YG, Uribe-Carvajal S, Sampedro JG (2009). Trehalose-mediated thermal stabilization of glucose oxidase from *Aspergillus niger*. J. Biotechnol..

[55] Koubaa S, Bremer A, Hincha DK, Brini F (2019). Structural properties and enzyme stabilization function of the intrinsically disordered LEA_4 protein TdLEA3 from wheat. Sci. Rep..

[56] Lv A (2018). Characterization of dehydrin protein, CdDHN4-L and CdDHN4-S, and their differential protective roles against abiotic stress in vitro. BMC Plant Biol..

[57] Liu J, Dai.  (2022). Expression, purification, and preliminary protection study of dehydrin PicW1 from the biomass of *Picea wilsonii*. Front. Bioeng. Biotechnol..

[58] Goyal K, Walton LJ, Tunnacliffe A (2005). LEA proteins prevent protein aggregation due to water stress. Biochem..

[59] Lin C, Thomashow MF (1992). A cold-regulated Arabidopsis gene encodes a polypeptide having potent cryoprotective activity. Biochem. Biophys. Res. Commun..

[60] Brini F (2010). Wheat dehydrin DHN-5 exerts a heat-protective effect on beta-glucosidase and glucose oxidase activities. Biosci. Biotechnol. Biochem..

[61] Drira M (2013). The K-segments of the wheat dehydrin DHN-5 are essential for the protection of lactate dehydrogenase and β-glucosidase activities in vitro. Mol. Biotechnol..

[62] Gasteiger, E. *et al*. The Proteomics protocols handbook (ed. Walker J.M.) 571–607 (Humana Press, 2005).

[63] Oates ME (2013). D2P2: Database of disordered protein predictions. Nucleic Acids Res..

[64] Jones DT, Cozzetto D (2015). DISOPRED3: Precise disordered region predictions with annotated protein-binding activity. Bioinformatics.

[65] Bailey TL, Johnson J, Grant CE, Noble WS (2015). The MEME suite. Nucleic Acids Res..

[66] Jones DT (1999). Protein secondary structure prediction based on position-specific scoring matrices. J. Mol. Biol..

[67] Nugent T, Jones DT (2009). Transmembrane protein topology prediction using support vector machines. BMC Bioinform..

[68] Hallgren, J. *et al*. DeepTMHMM predicts alpha and beta transmembrane proteins using deep neural networks. *bioRxiv* 2022.04.08.487609 (2022).

[69] Tsirigos KD, Peters C, Shu N, Käll L, Elofsson A (2015). The TOPCONS web server for combined membrane protein topology and signal peptide prediction. Nucleic Acids Res..

[70] Jumper J (2021). Highly accurate protein structure prediction with AlphaFold. Nature.

[71] Katoh K, Standley DM (2013). MAFFT Multiple sequence alignment software version 7: improvements in performance and usability. Mol. Biol. Evol..

[72] Letunic I, Bork P (2021). Interactive tree of life (iTOL) v5: An online tool for phylogenetic tree display and annotation. Nucl. Acids Res..

