# Supplementary material for: Enzyme stabilization and thermotolerance function of the intrinsically disordered LEA2 proteins from date palm
Source: Sci Rep. 2023 Jul 23;13:11878. doi: 10.1038/s41598-023-38426-w (PMC10363547; doi:10.1038/s41598-023-38426-w)
Supplement: Supplementary file 1 — Supplementary Figures. [file 41598_2023_38426_MOESM1_ESM.docx]

**Enzyme stabilization and thermotolerance function of the intrinsically disordered LEA2 proteins from date palm**

Mughair Abdul Aziz^1†^, Miloofer Sabeem^1†^, M. Sangeeta Kutty^2^, Shafeeq Rahman^1^, Maitha Khalfan Alneyadi^1^, Alia Binghushoom Alkaabi^1^, Eiman Saeed Almeqbali^1^, Faical Brini^3^, Ranjit Vijayan^4^, Khaled Masmoudi^1*^

^1^Department of Integrative Agriculture, College of Agriculture and Veterinary Medicine, United Arab Emirates University, Al‑Ain, Abu‑Dhabi, UAE

^2^Department of Vegetable Science, College of Agriculture, Kerala Agricultural University, Vellanikkara. Thrissur-680656, India

^3^Biotechnology and Plant Improvement Laboratory, Centre of Biotechnology of Sfax (CBS)/ University of Sfax, Sfax, Tunisia

^4^Department of Biology, College of Science, United Arab Emirates University, Al‑Ain, Abu‑Dhabi, UAE

**^†^**The authors contributed equally into this work

^*^Corresponding author: Khaled Masmoudi

Email: khaledmasmoudi@uaeu.ac.ae

**Supplementary figures**


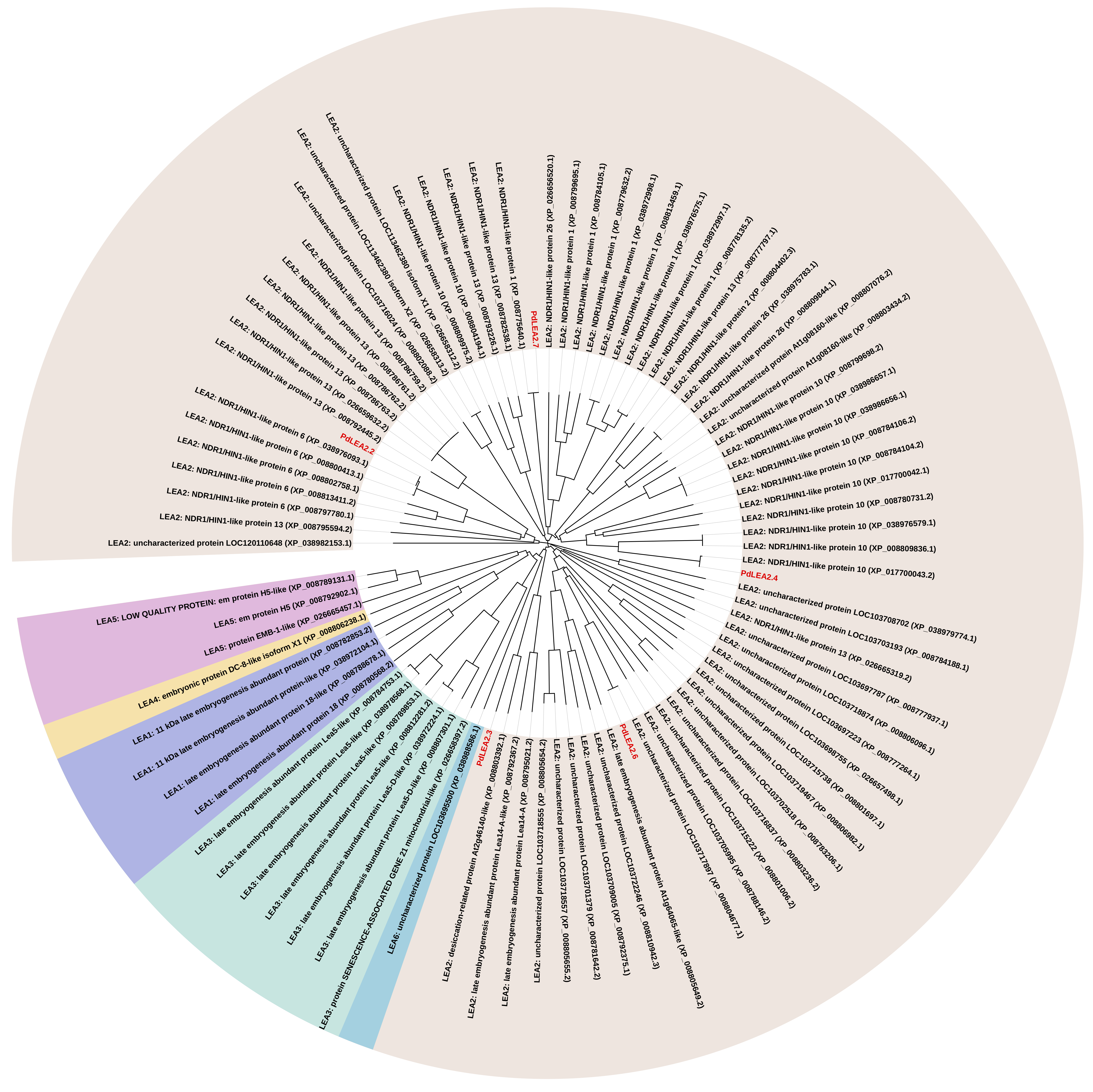


**Supplementary Figure S1.** Phylogenetic tree of PdLEA2 proteins.


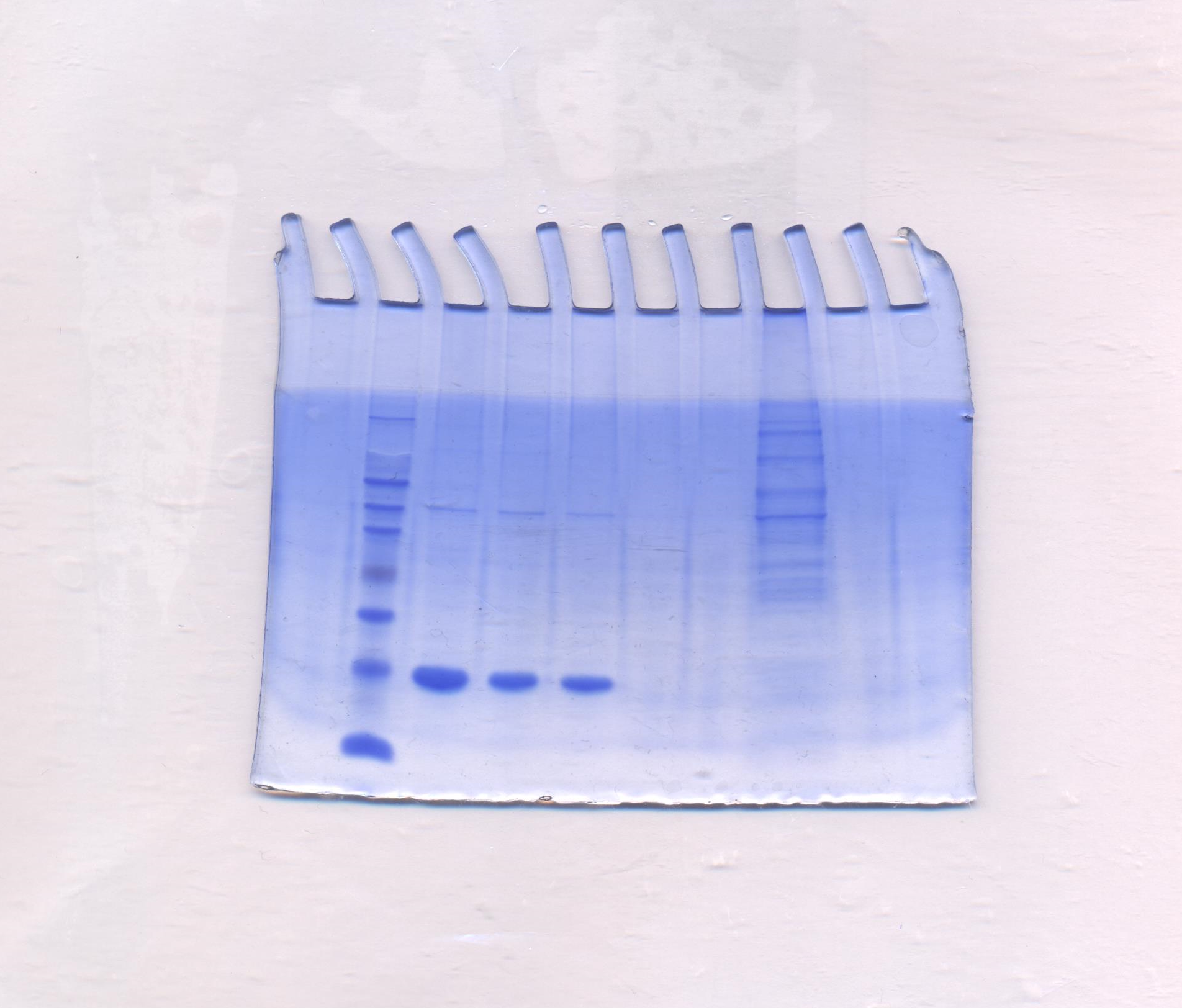


**MW Lea2-4 Lea2-3 Lea2-2 C**

**A**

**SDS-PAGE**


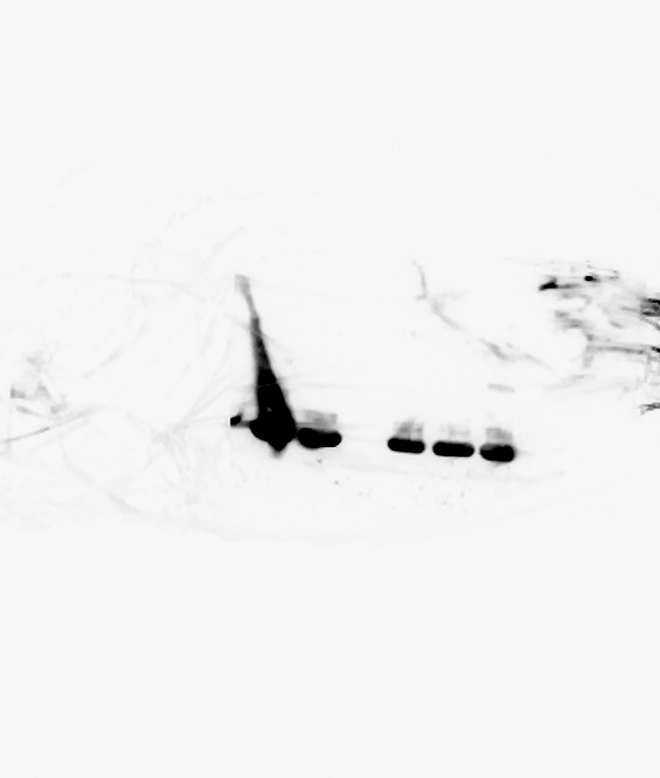


**C Lea2.4 Lea2.3 Lea2.2**

**B**

**Western blotting**

**Supplementary Figure S2.** SDS-PAGE and Western blotting of PdLEA2 proteins.
