# Supplementary material for: Enzyme stabilization and thermotolerance function of the intrinsically disordered LEA2 proteins from date palm
Source: Sci Rep. 2023 Jul 23;13:11878. doi: 10.1038/s41598-023-38426-w (PMC10363547; doi:10.1038/s41598-023-38426-w)
Supplement: Supplementary file 2 — Supplementary Tables. [file 41598_2023_38426_MOESM2_ESM.docx]

**Enzyme stabilization and thermotolerance function of the intrinsically disordered LEA2 proteins from date palm**

Mughair Abdul Aziz^1†^, Miloofer Sabeem^1†^, M. Sangeeta Kutty^2^, Shafeeq Rahman^1^, Maitha Khalfan Alneyadi^1^, Alia Binghushoom Alkaabi^1^, Eiman Saeed Almeqbali^1^, Faical Brini^3^, Ranjit Vijayan^4^, Khaled Masmoudi^1*^

^1^Department of Integrative Agriculture, College of Agriculture and Veterinary Medicine, United Arab Emirates University, Al‑Ain, Abu‑Dhabi, UAE

^2^Department of Vegetable Science, College of Agriculture, Kerala Agricultural University, Vellanikkara. Thrissur-680656, India

^3^Biotechnology and Plant Improvement Laboratory, Centre of Biotechnology of Sfax (CBS)/ University of Sfax, Sfax, Tunisia

^4^Department of Biology, College of Science, United Arab Emirates University, Al‑Ain, Abu‑Dhabi, UAE

**^†^**The authors contributed equally into this work

^*^Corresponding author: Khaled Masmoudi

Email: khaledmasmoudi@uaeu.ac.ae

**Supplementary tables**

Table S1: NCBI Accession of PdLEA2 mRNA, protein sequences, and chromosomal location based on date palm Barhee BC4 genome assembly

| **PdLEA2 protein** | **NCBI mRNA Accession** | **NCBI Protein Accession** | **Chromosome** |
| --- | --- | --- | --- |
| PdLEA2.2 | OQ348181.1 | WCF34208.1 | - |
| PdLEA2.3 | OQ348184.1 | WCF34211.1 | 7 |
| PdLEA2.4 | OQ348183.1 | WCF34209.1 | 14 |
| PdLEA2.6 | OQ348182.1 | WCF34210.1 | 3 |
| PdLEA2.7 | OQ348185.1 | WCF34212.1 | 2 |

Table S2: Primers sequences used to isolate the *LEA2* genes from date palm

| **Primers** | **Sequences (5’ – 3’)** |
| --- | --- |
| *PdLEA2.2* Fw | ATGGCCGAACAGCAGAGA |
| *PdLEA2.2* Rv | TCAGAGCTTCAACCTAAACT |
| *PdLEA2.3* Fw | ATGGCATCCTCTGATAAGC |
| *PdLEA2.3* Rv | TTAGCATACCTCATCATCATC |
| *PdLEA2.4* Fw | ATGGGAAGAACTTATTACGG |
| *PdLEA2.4* Rv | TTAGTAATCTACAACATCGCA |
| *PdLEA2.6* Fw | ATGAAGGAATCAGGCGACT |
| *PdLEA2.6* Rv | TCAGAGCTTGGTCTTCTG |
| *PdLEA2.7* Fw | ATGCCTCAAGGCACCACGA |
| *PdLEA2.*7 Rv | TCAGATGTCGACGTCGCAG |

Table S3: Oligonucleotides sequence of *PdLEA2* genes used in real-time PCR expression analysis

| **Primers** | **Nucleotide sequences (5’ – 3’)** | **Accession no.** |
| --- | --- | --- |
| Date palm Actin gene |  | XM_008778129.4 |
| Actin F | GCGATTCAGGCAGTTCTTTC |  |
| Actin R | AATTTCCCGTTCTGCAGTTG |  |
| *PdLEA2.2* gene |  | OQ348181 |
| *PdLEA2.2* F | CGGAGTTTTATCAGGGGCAC |  |
| *PdLEA2.2* R | TGAGACTCCCAAGCTTCACC |  |
| *PdLEA2.3* gene |  | OQ348184 |
| *PdLEA2.3* F | ACCCTGCCTTTGGAGAAGAA |  |
| *PdLEA2.3* R | ATGCATTGAGTCCCAGGTCA |  |
| *PdLEA2.4* gene |  | OQ348183 |
| *PdLEA2.4* F | AGTCTTGAAGGGGAACTCGG |  |
| *PdLEA2.4* R | CCTGAAGTTCGATGAGCAGC |  |

Table S4: Primers corresponding to *PdLEA2* genes with EcoRI and BamHI restriction sites added

| **Primers** | **Sequences (5’ – 3’)** |
| --- | --- |
| PdLEA2.2BamHI Fw | GGGATCCATGGCCGAACAGCAGAGA |
| PdLEA2.2EcoRI Rv | GGAATTCTCAGAGCTTCAACCTAAAC |
| PdLEA2.3BamHI Fw | GGGATCCATGGCATCCTTTGATAAGC |
| PdLEA2.3EcoRI Rv | GGAATTCTTAGCATACCTCATCATC |
| PdLEA2.4BamHI Fw | GGGATCCATGGGAAGAACTTATTACGG |
| PdLEA2.4EcoRI Rv | GGAATTCTTAGTAATCTACAACATC |
